# A Rare Case of Primary Gastric HIV-Associated Peripheral T-Cell Lymphoma: Relapsed Disease Treated With Pemetrexed

**DOI:** 10.4021/wjon708w

**Published:** 2013-09-27

**Authors:** Jun Gong, Maria L. Delioukina, Andrew E. Hendifar

**Affiliations:** a Department of Internal Medicine, Cedars-Sinai Medical Center, 8700 Beverly Blvd, #5512, Los Angeles, CA 90048, USA; b Blood and Marrow Transplant Program, Samuel Oschin Cancer Center, Cedars-Sinai Medical Center, 8700 Beverly Blvd, AC 1076, Los Angeles, CA 90048, USA; c Hematology and Oncology, Samuel Oschin Cancer Center, Cedars-Sinai Medical Center, 8700 Beverly Blvd, AC 1042C, Los Angeles, CA 90048, USA

**Keywords:** Peripheral T-cell lymphoma, HIV/AIDS, Pemetrexed

## Abstract

We present a case of a patient with HIV/AIDS who presented with abdominal pain and melena and was found to have gastric peripheral T-cell lymphoma (PTCL). He was treated with 6 cycles of EPOCH with a complete response. Within 3 months, he had central nervous system (CNS) and soft-tissue relapse. He was subsequently treated with 3 cycles of intravenous pemetrexed and experienced a second complete response. To our knowledge, there are fewer than 100 reports of HIV-associated PTCL worldwide as of 2010, and among these cases involvement of the stomach as the primary site of extranodal disease is exceptionally rare. The disease carries a poor prognosis and current standard therapies highlight the importance of HIV/AIDS disease control and the need for more studies on therapies and the role of transplantation in refractory or relapsed HIV-associated PTCL.

## Introduction

HIV-associated PTCL is comprised of a heterogenous group of aggressive neoplasms. The most common primary site of involvement is often extranodal, and a majority of cases present with B symptoms, advanced stage, low CD4 counts, and high HIV viral loads. Diagnosis is made by biopsy showing positive staining for T-cell antigens in the absence of B-cell antigens. The mainstay of therapy involves most commonly CHOP or CHOP-like regimens and antiretroviral therapy, though the overall prognosis is very poor. Several clinical trials involving novel agents are underway to address refractory or relapsed disease. The role of transplantation in refractory or relapsed disease is less clear though certain subgroups of patients with PTCL may benefit more than others.

## Case Report

A 33-year-old male with a history of HIV/AIDS presented to the emergency department with intermittent right upper quadrant abdominal pain, fevers, hematemesis and melena for several weeks. Vitals showed a temperature of 37.1 °C, heart rate of 148, respiratory rate of 16, blood pressure of 111/63, and oxygen saturation of 100% on room air. Physical exam revealed a thin male in no apparent distress. His exam was unremarkable aside from tachycardia with a regular rhythm on cardiac exam. Pertinent negatives included a benign abdominal exam and absence of any significant lymphadenopathy or dermatologic findings. Laboratories were notable for a hemoglobin of 6.5 g/dL, mean corpuscular volume (MCV) of 86.1 fL, and platelet count of 6,000 with an otherwise unremarkable differential, LDH of 185 U/L, ESR of 34 MM, CRP of 6.58 mg/dL, absolute CD4 count of 41 CMM, and a positive EIA and Hemoccult for stool occult blood.

**Figure 1:**
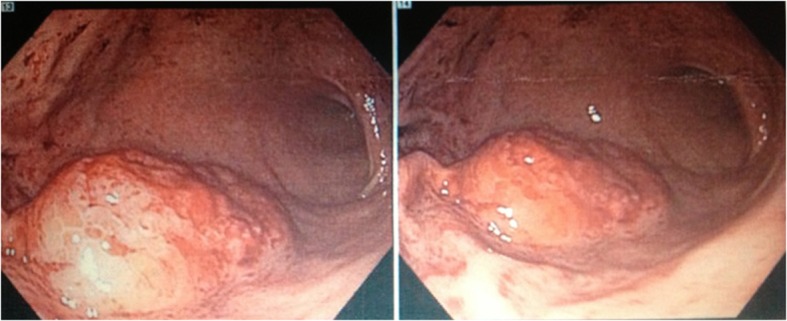
Esophagogastroduodenoscopy (EGD) revealing a large mass located in the gastric antrum with biopsy later showing the presence of clusters of malignant lymphoid cells with staining characteristics consistent with peripheral T-cell lymphoma.

An esophagogastroduodenoscopy (EGD) was performed which revealed a large gastric antral mass that was biopsied (Fig. 1) as well as multiple bleeding Dieulafoy lesions in the gastric fundus that were subsequently hemoclipped. The biopsy report showed clusters of large, monomorphic, malignant lymphoid cells with 1). positive staining for CD3, CD4, CD8, and MUM-1; 2). a high proliferation rate (95%) by Ki-67; and 3). negative staining for CD10, CD20, CD30, CD56, EBER, ALK-1, and TIA-1. These findings were consistent with peripheral T-cell lymphoma but excluded the diagnosis of ALK-1 positive and negative anaplastic large cell lymphoma, extranodal NK/T-cell lymphoma, and cytotoxic T-cell lymphoma. A staging CT scan showed an approximately 2 × 1.9 cm soft-tissue mass inseparable from the right psoas but without any significant mediastinal, hilar, or axillary lymphadenopathy. The remainder of his staging work-up including bone marrow biopsy, CSF, and further imaging would return negative for involvement by lymphoma.

The patient would complete 6-cycles of EPOCH (etoposide, prednisone, vincristine, cyclophosphamide, and doxorubicin) and intrathecal (IT) methotrexate. A surveillance PET-CT would later show metabolic activity consistent with treatment effect on the bone marrow but otherwise absence of activity to suggest recurrence of lymphoma. The patient would later return with right lower extremity/lower back radiculopathy and left upper extremity swelling with an approximate 4 × 7 cm area of erythema that was tender and warm to palpation.

**Figure 2:**
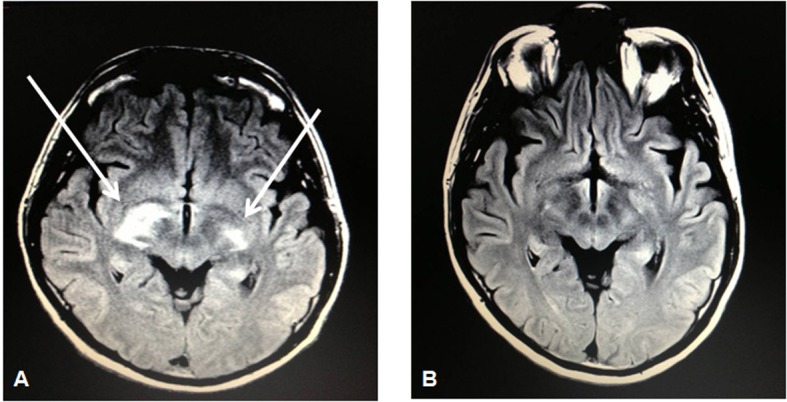
MRI brain showing bilateral signal enhancement (more on the right than left side) within the lateral subthalamus, midbrain, and internal capsule (A) followed by resolution of such lesions on a repeat MRI brain 5 months later (B) after 3 cycles of intravenous pemetrexed 900 mg/m^2^.

Laboratories were notable for an absolute CD4 count of 23 CMM and HIV viral load of 76,000 copies/mL despite having been on antiretroviral therapy since his HIV diagnosis 4 years earlier. Subcutaneous tissue biopsy of the left triceps showed clusters of large malignant lymphoid cells with staining characteristics consistent with PTCL as before. A repeat staging CT scan showed a 3.4 × 2.1 cm L5-level mass posterior to the right psoas. An MRI of the brain would show bilateral signal enhancement within the lateral subthalamus, midbrain, and internal capsule (Fig. 2). CSF analysis would show the presence of atypical lymphoid cells suspicious for lymphoma. Bone marrow biopsy, however, would show an absence of involvement by lymphoma.

The patient subsequently received high-dose intravenous (IV) methotrexate (8 g/m^2^) for his relapsed peripheral T-cell lymphoma (stage IV) that originally manifested as a gastric mass. Due to extremely poor elimination following the methotrexate administration, he would instead complete 3 cycles of IV pemetrexed 900 mg/m^2^ (in 3-week cycles) that was well tolerated as he experienced only a grade I nausea as an adverse event. Subsequent restaging PET-CT and MRI of the brain would show a complete radiographic response (Fig. 2). At the time of this report, he is scheduled to undergo autologous stem cell transplantation in the setting of his second complete remission.

## Discussion

Peripheral T-cell lymphoma (PTCL) is a subtype of non-Hodgkin lymphoma (NHL) and comprised of a heterogeneous group of neoplasms [1]. In Western parts of the world, PTCL accounts for approximately 5 to 10 percent of all cases of NHL, and this percentage is higher in Asian countries [2]. In the United States, the incidence of PTCL is about less than 1 case per 100,000 individuals [1]. The incidence of PTCL has been shown to increase by 15-fold when associated with HIV infection [3]. Despite this association, however, HIV-associated PTCL remains an exceptionally rare disease with fewer than 100 cases reported in the literature worldwide as of 2010 [4]. Of these cases, the most common subtypes of HIV-associated PTCL, in order of decreasing frequency, are PTCL unspecified (PTCL-U), anaplastic large cell lymphoma (ALCL), NK/T-cell lymphoma (NKTCL), T-cell primary central nervous system lymphoma (PCNSL), adult T-cell leukemia/lymphoma (ATLL), angioimmunoblastic T-cell lymphoma (AITL), T-cell primary effusion lymphoma (PEL), enteropathy-like T-cell lymphoma (ELTL), and subcutaneous panniculitis-like T-cell lymphoma (SPTCL) [5]. The median age at presentation is 38 years (more than 98% of cases are under 60 years of age) with a 4:1 male-to-female preponderance, and a majority of cases occur in Caucasians, Hispanics, and Blacks [4, 5].

The clinical presentation of HIV-associated PTCL, like other AIDS-related lymphomas, is characterized by extranodal disease as the primary site of involvement in a majority (83%) of cases [5, 6]. In fact, only 17% of cases have been shown to be exclusively nodal at the time of presentation [5]. The most common sites of extranodal disease are bone marrow (31%), head and neck (23%), lungs (15%), GI tract (14%), skin (9%), and CNS (9%) [5]. In a separate study, involvement of the stomach is among the least common sites of extranodal disease [7]. A majority of cases also tend to present with advanced disease (75% with Ann Arbor stage III or IV), B symptoms (66% of cases), and elevated LDH levels (68% of cases) [4-7]. With respect to HIV status, the median absolute CD4 count was 137 CMM (71% of cases present with counts less than 200 CMM), median HIV viral load was 343,787 copies/mL, median time from detection of HIV infection to PTCL diagnosis was 55 months, and 25% of cases were on HAART at the time of diagnosis of PTCL [4].

The definitive diagnosis of PTCL relies on biopsy of the involved site. The histopathologic features of HIV-associated PTCL include the presence of malignant lymphoid cells that stain positively for T-cell antigens such as CD3, CD4, CD5, CD8, CD43, and/or CD56 but lack staining for B-cell antigens such as CD20 [7]. More specifically, positive staining for ALK and CD30 is associated with ALCL though ALK-negative cases also exist, EBV-positivity via EBV-encoded RNA (EBER) and CD56-positivity is associated with extranodal NKTCL though EBV-positivity has been associated with other PTCL subtypes as well, and positive staining for CD10 is associated with AITL [4, 5, 7]. Much rarer subtypes of PTCL such as ATLL, ELTL, and PEL have been associated with HTLV-1-positivity, celiac disease and/or dermatitis herpetiformis, and HHV8-positivity, respectively [4, 5, 7]. Lastly, HIV-associated PTCL is associated with relative high expression of Ki-67 and detection of T-cell receptor (TCR) gene rearrangements [4, 5, 7].

Despite results showing that 53% of cases have an ECOG score of 0-1, 56% of cases having an International Prognostic Index (IPI) score of 0-1, and 49% of cases having a Prognostic Index for PTCL (PIT) score of 0-1, HIV-associated PTCL carries a poor prognosis [4]. The median overall survival (OS) has been shown to range from 6-12 months with a 5-year OS of 32% [4, 5, 7]. Negative prognostic indicators include absolute CD4 counts less than 200 CMM, ECOG scores greater than or equal to 2, and advanced stage [4, 5]. The use of HAART has been shown to be a positive prognostic indicator [8]. The majority of cases of HIV-associated PTCL (63%) die from progression of disease [4].

The most common forms of induction therapy for HIV-associated PTCL include cyclophosphamide, doxorubicin, vincristine, and prednisone (CHOP) or CHOP-like regimens and aggressive antiretroviral therapy [4, 5, 7]. The overall response rate (ORR) to chemotherapy has been shown to be 64% with 43% experiencing a complete response (CR) and 21% experiencing a partial response (PR) [4]. Although studies regarding therapies in relapsed or refractory HIV-associated PTCL are lacking, novel agents including denileukin diftitox, romidepsin and belinostat, pralatrexate, lenalidomide, and bortezomib are demonstrating relatively favorable response rates in their respective clinical trials [8].

In particular, the use of the antifolate pralatrexate in the PROPEL trial produced an ORR of 29% with a median duration of response of 10.1 months in 111 patients with relapsed or refractory PTCL [8]. Data is limited in evaluating the efficacy of pemetrexed in those with relapsed or refractory HIV-associated PTCL. However, a recent study demonstrated that pemetrexed, an antifolate that inhibits thymidylate synthase and glycinamide ribonucleotide formyltransferase in addition to dihydrofolate reductase, produced an ORR of 58.3%, CR rate of 16.7%, and median progression free survival (PFS) of 2.5 months in 12 patients with secondary CNS lymphoma treated with IV pemetrexed 900 mg/m^2^ every 3 weeks [9]. The role of transplantation in PTCL is highlighted by studies that demonstrate greater benefit following autologous transplantation in those who achieved initial remission (5-year OS rate of 76%) than in those with relapsed disease (5-year OS rate of 36%) while outcomes appear more favorable with allogeneic transplantation in those with refractory or relapsed disease [8]. Nevertheless, the role of autologous transplantation in subsequent remissions after the first is less clear, and further studies are needed to better define subpopulations of HIV-associated PTCL patients who will benefit from allogeneic versus autologous transplantation [8].
